# Supplementary material for: Enhancing the Properties of Water-Soluble Copolymer Nanocomposites by Controlling the Layer Silicate Load and Exfoliated Nanolayers Adsorbed on Polymer Chains
Source: Polymers (Basel). 2023 Mar 12;15(6):1413. doi: 10.3390/polym15061413 (PMC10056508; doi:10.3390/polym15061413)
Supplement: Supplementary file 1 [file polymers-15-01413-s001.zip › polymers-2163532-supplementary.pdf]

Supplementary Materials

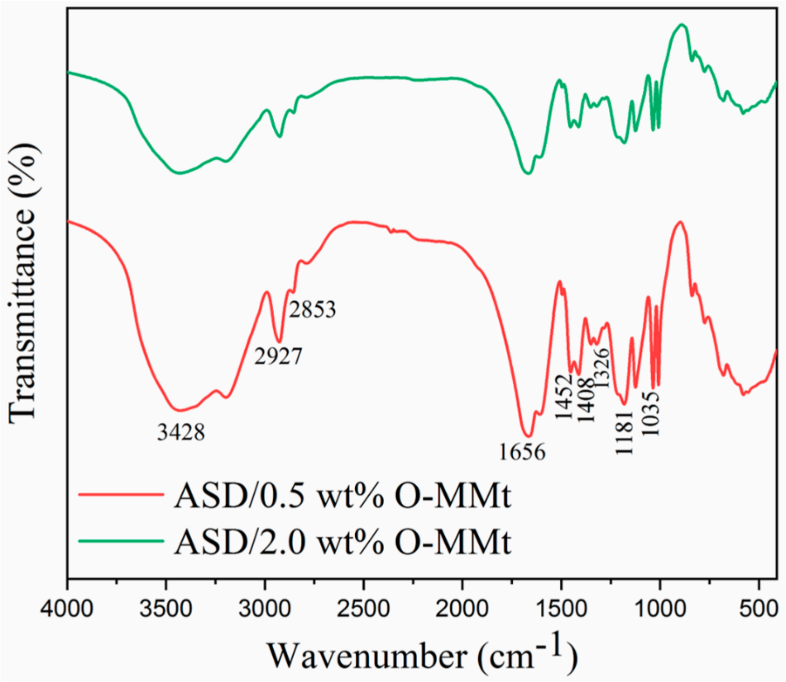

Figure S1. FTIR spectra of ASD/0.5 wt% O-MMt and ASD/2.0 wt% O-MMt.

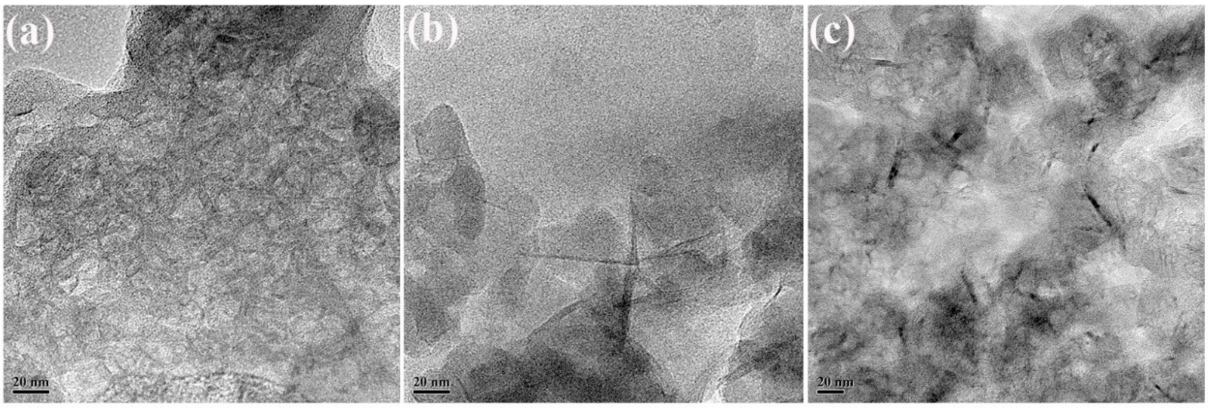

Figure S2. TEM images of (a)ASD, (b)ASD/0.5 wt% O-MMt and (c)ASD/2.0 wt% O-MMt.

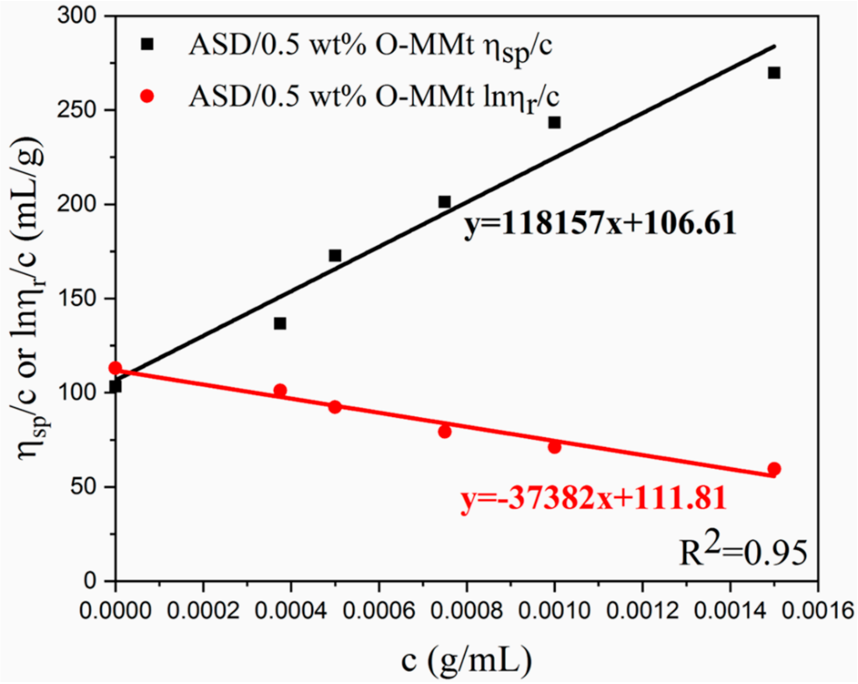

Figure S3. Relationship between η_{sp}/c or $\ln\eta_r/c$ and the ASD/0.5 wt% O-MMt concentration.

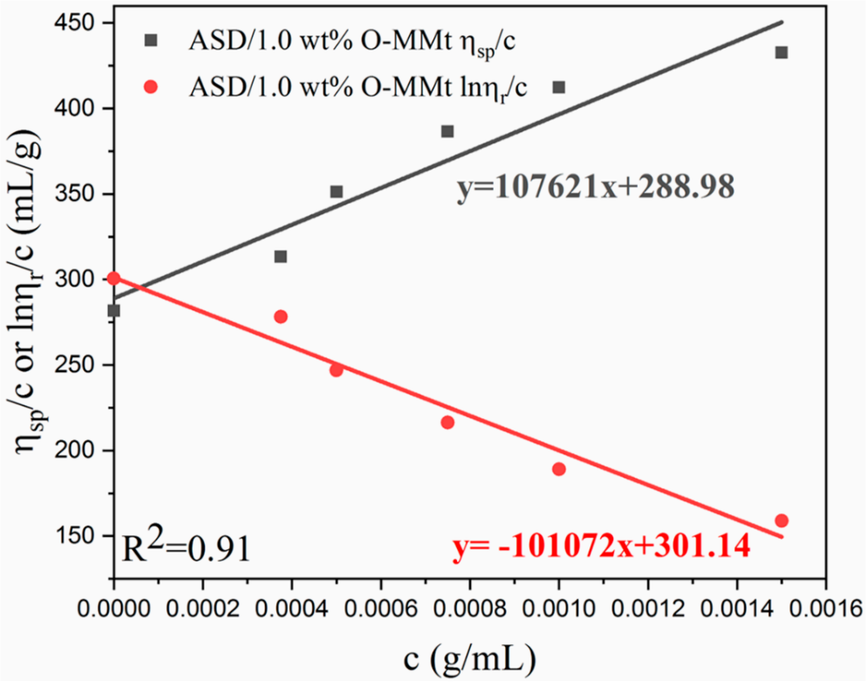

Figure S4. Relationship between η_{sp}/c or $\ln\eta_r/c$ and the ASD/1.0 wt% O-MMt concentration.

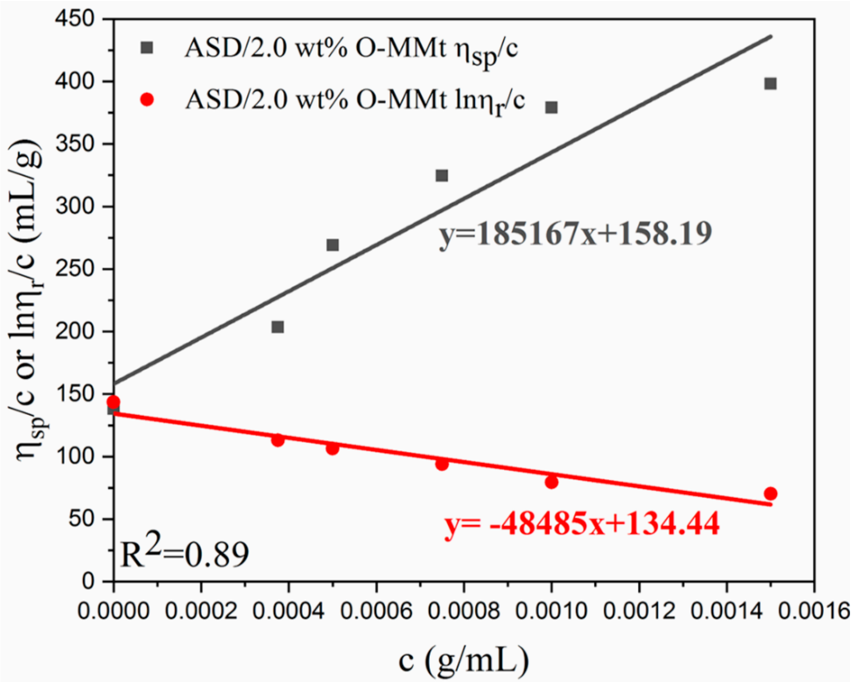

Figure S5. Relationship between η_{sp}/c or $\ln\eta_r/c$ and the ASD/2.0 wt% O-MMt concentration.

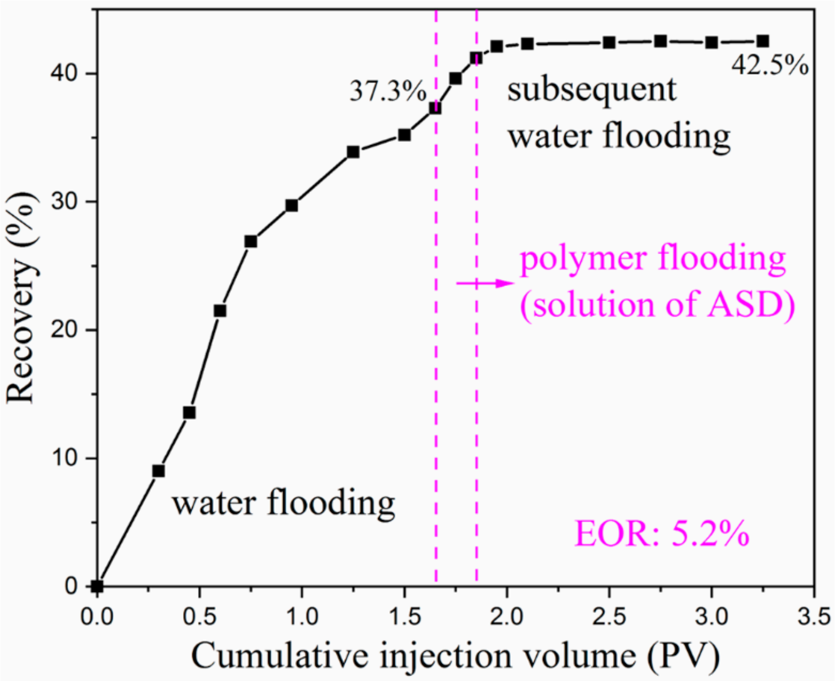

Figure S6. EOR experiments using ASD under laboratory conditions.

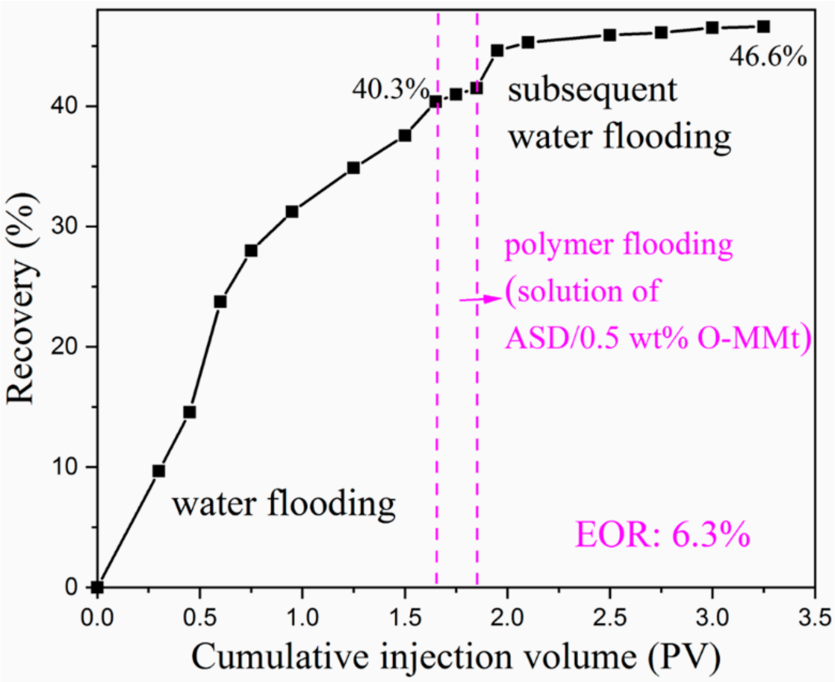

Figure S7. EOR experiments using ASD/0.5 wt% O-MMt under laboratory conditions.

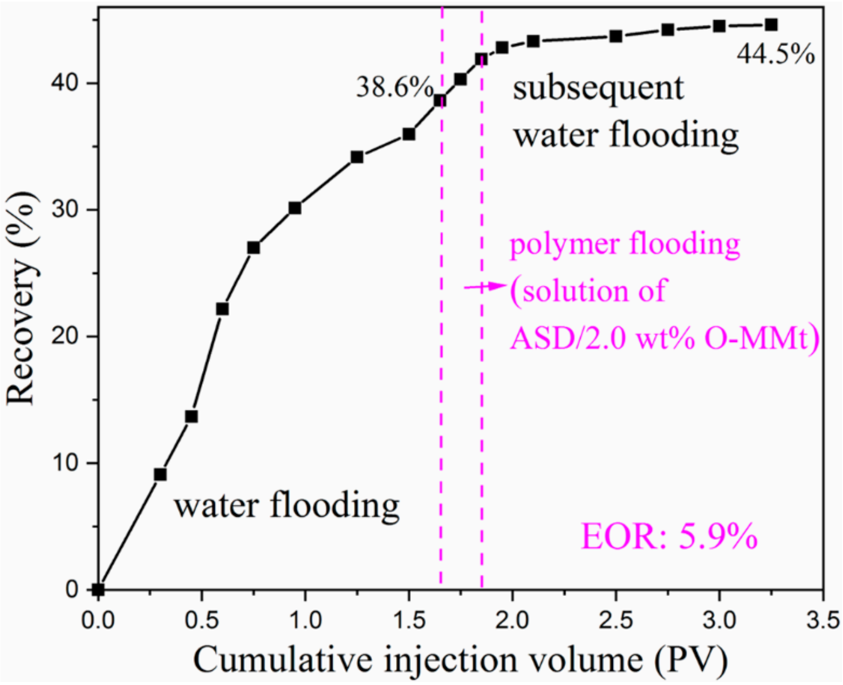

Figure S8. EOR experiments using ASD/2.0 wt% O-MMt under laboratory conditions.
